# Perfusion Microfermentor Integrated into a Fiber Optic Quasi-Elastic Light Scattering Sensor for Fast Screening of Microbial Growth Parameters

**DOI:** 10.3390/s19112493

**Published:** 2019-05-31

**Authors:** Marco César Prado Soares, Franciele Flores Vit, Carlos Kenichi Suzuki, Lucimara Gaziola de la Torre, Eric Fujiwara

**Affiliations:** 1 Laboratory of Photonic Materials and Devices, School of Mechanical Engineering, University of Campinas, São Paulo 13083-860, Brazil; suzuki@fem.unicamp.br (C.K.S.); fujiwara@fem.unicamp.br (E.F.); 2 Laboratory of Advanced Development of Nano and Biotechnology, School of Chemical Engineering, University of Campinas, São Paulo 13083-852, Brazil; franciele.floresvit@gmail.com (F.F.V.); ltorre@g.unicamp.br (L.G.d.l.T.)

**Keywords:** fiber optic sensor, microfermentor, quasi-elastic light scattering, microbial growth screening, biological monitoring

## Abstract

This research presents a microfermentor integrated into an optical fiber sensor based on quasi-elastic light scattering (QELS) to monitor and swiftly identify cellular growth kinetic parameters. The system uses a 1310 nm laser light that is guided through single-mode silica optical fibers to the interior of perfusion chambers, which are separated by polycarbonate membranes (470 nm pores) from microchannels, where a culture medium flows in a constant concentration. The system contains four layers, a superior and an inferior layer made of glass, and two intermediate poly(dimethylsiloxane) layers that contain the microchannels and the perfusion chambers, forming a reversible microfluidic device that requires only the sealing of the fibers to the inferior glass cover. The QELS autocorrelation decay rates of the optical signals were correlated to the cells counting in a microscope, and the application of this microsystem to the monitoring of alcoholic fermentation of *Saccharomyces cerevisiae* resulted in the kinetic parameters of K_M_ = 4.1 g/L and μ_m_ = 0.49 h^−1^. These results agree with both the data reported in the literature and with the control batch test, showing that it is a reliable and efficient biological monitoring system.

## 1. Introduction

The main goals of monitoring and controlling a bioprocess and its intrinsic kinetics are to keep the adequate conditions for the biocatalyzer—the cells and microorganisms on fermentations—and to evaluate their concentration (denoted by X), which is the basic parameter of fermentation mathematical models [1]. However, these tasks are usually accomplished by techniques unsuitable for automatic control. Traditional analytical methods rely on the use of very expensive and non-portable instruments, such as epifluorescence microscopes, centrifuges, spectrophotometers, etc. [2]. Moreover, many traditional measurements, such as, for instance, the dry mass, are based on manual and time-consuming procedures [1]. In most cases, the measurement techniques rely on the quantification of a specific property, either physical (e.g., variation of the medium’s refractive index, viscosity, or electrical conductivity [3]) or biochemical (concentration of proteins, carbohydrates, DNA or RNA, for example [4]), which are posteriorly correlated to the concentration of cells by an appropriate model.

Over the last two decades, a promising methodology has emerged for the study of important fluidic chemical and biochemical systems, namely microfluidics, commonly defined as the science which studies the manipulation of small amounts of fluids flowing through micrometric channels in predominantly laminar regimes without turbulence [5]. It presents many advantages compared to macroscale processes, such as the low consumption of reagents, the creation of biomimetic environments, and the possibility of minimum contact manipulation of pathogenic cells, reducing the risk of contamination [6,7,8]. Despite these advantages, microfluidic systems still lack instrumentation and automation alternatives [9,10].

Therefore, the integration of optical fiber sensors to microfluidic components is promising to provide simpler alternatives to the measurement of the flow and to the assessment of other important biochemical parameters [10], such as the total dissolved oxygen in a microfluidic bioreactor [11]. Optical fibers seem to be very attractive for the application in biochemical microsystems since they are biocompatible and immune to electromagnetic interference. Moreover, optical fibers demonstrate chemical and thermal stability [12] that, along with reduced fabrication costs, makes them suitable for the mass-fabrication of devices [13].

Considering the difficulties found in the assessment of biochemical microsystems and the advantages of optical fibers, we propose a monitoring alternative based on the fabrication of a microfluidic perfusion fermentor instrumented with a fiber optic quasi-elastic light scattering sensor (FOQELS). The system is used for the direct monitoring of the cell concentration inside reaction chambers, leading to the fast screening of the microbial growth kinetics and is aimed to define the best processing conditions, with replicates obtained from the same experiment. The device takes advantage of the low superficial area required for optical systems, which allows integration to the micro dimensions and the in-situ monitoring with low interference in the cellular medium and metabolism [14]. 

Although the optical monitoring of microfluidics has been studied previously, there are only a few studies in the literature demonstrating the specific application of the quasi-elastic light scattering and the Fresnel reflectometry for the evaluation and quantification of cells inside the devices. There is a report regarding the scattering detection on a medium containing cells of *Escherichia coli*, with evidence that the phenomenon was proportional to the total mass [15], and another about the fabrication of a flow cytometer (a system used for counting and dimensioning cells). It was a complex device that relied on polymeric solutions to generate the cells elasto-inertial focusing effect and on two different lasers guided through microstructured optical fibers to three photodetectors, one of which quantified the total scattering of both light sources [16]. Finally, in a previous study with fermentation macrosystems comprised of only water, sucrose, ethanol, and cellular mass, we verified correspondence between the scattering evaluated with a sensor based on single-mode optical fibers (SMFs) and the behavior expected from the theoretical kinetic curves [17].

The results presented here using the microfermentor were validated by comparing them with the analysis by optical microscopy. Unlike the counting with the cytometer, this visual cell counting procedure does not require the perfect alignment of the cells on a flowing stream, and the quality of the results can be improved by the addition of dyes, as detailed in Section 2 [1,2,16].

The counting methodologies lead to the obtention of concentration results in the number of cells per volume, whereas the dry-mass analysis obtains the corresponding values in mass per volume. All of the mathematical symbols used for the data interpretation are summarized in Table 1, where they are given in the order they are introduced in the text.

## 2. Materials and Methods

### 2.1. Microfermentor Design and Fabrication

A system that is suitable for the growth of many microorganisms, especially more refined cultures, is the perfusion reactor, also called the “internal biomass feedback”, which basically consists of a bioreactor containing a mechanical device (e.g., a filter) capable of physically retaining the cells, while allows the continuous addition of substrates (the cells’ nutrients, commonly denoted by S) and the removal of products, consumed culture media, and toxic metabolites [4].

The microfermentor proposed in this work is an adaptation of this concept to microfluidics and is made of four layers, a superior borosilicate glass cover, two layers of poly(dimethylsiloxane), PDMS, and an inferior glass cover (covers’ thicknesses of 1 mm). The superior cover of glass contains holes for the admission and removal of liquid, whereas the inferior layer presents holes for the introduction of the SMFs, which are posteriorly sealed to the glass with epoxy resin in order to be maintained in the correct positions and to prevent leakages, misalignment, loss of contact between the fluid and the fibers, and optical losses by fiber macrobendings. 

The PDMS layers were fabricated by laser ablation of a laminated PDMS sheet with 0.50 mm thickness (Stockwell Elastomerics, Philadelphia, PA, USA) using a CO_2_ laser (λ = 10.6 μm, power fixed in 10 W, L-Solution 100, Gravograph, Northmont Parkway, Duluth, GA, USA). The bottom layer of the PDMS contains three parallel perfusion chambers for the inoculation of the microorganisms to be analyzed. The fact that the chambers are positioned above the fibers improves the contact between the SMFs and the liquid and prevents the interruption of the signal by flow fluctuations or by the passage of bubbles.

The inferior level is isolated from the PDMS top layer by polycarbonate membranes with pores with an average diameter of 470 nm (Whatman Nuclepore WHA 110407, GE Healthcare Life Sciences, Marlborough, MA, USA). The top level, in turn, contains three microchannels that were designed based on the “tree-shaped” concentration generator, being formed by the recombining of the microchannels flows [18]. This recombination and the presence of coil structures on the channels are responsible for the homogenization of the fluid that flows above the chambers, so the cells are submitted to an approximately constant value of substrate concentration S [19].

Even though the substrate supplies used in this research are generally obtained as homogeneous liquids, the coils were added for allowing the same microfermentor to be used for the analyses of the concentration gradients (application of different concentrations to each fluid [2]), or of non-homogeneous suspensions. 

**Figure 1:**
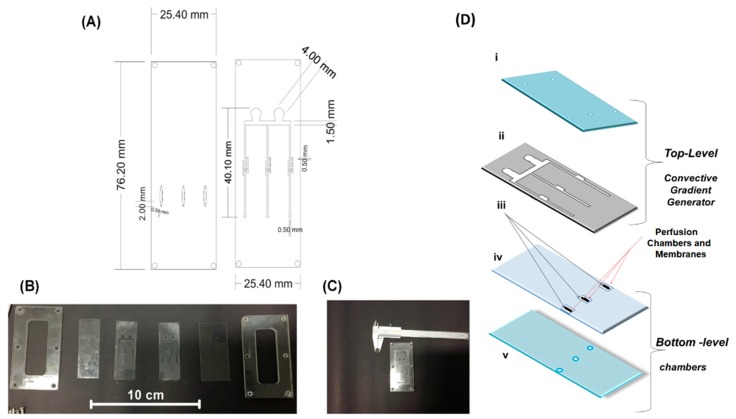
(**A**) Project of the two poly(dimethylsiloxane), PDMS, layers of the microfermentor; (**B**) layers and acrylic molds before the fermentor assembly; (**C**) microfermentor before the insertion and sealing of the optical fibers; (**D**) schematic diagram of the microfermentor: top-level (i) cover (glass), and (ii) microchannels (PDMS laminated); and bottom-level (iii) membranes (polycarbonate), (iv) intermediate sheet with holes (PDMS laminated), and (v) base (glass).

Before the system assembly, the four layers must be washed with neutral detergent and dried in a stove under 40 °C. Then, the layers were pressed on each other for adhesion forces to keep them together. Finally, acrylic molds were screwed externally to the four layers for maintaining the whole system fixed and with no leakages. A peristaltic pump was used for pumping the substrate solutions through the microchannels. The schematic drawings of the microfermentor, the layers before the system being put up together, the assembled system before the insertion and the sealing of the optical fibers, and the assembly sequence are shown in Figure 1.

This system with no fibers was also applied for visual monitoring in the control experiment, as shown in the following section.

### 2.2. Optical Fiber Sensor Design

The optical fiber analytical system used in this research is comprised of commercially available telecommunication devices. The light emitted by the laser diode (continuous wave, 1310 nm) is guided through the SMF to the liquid medium of the perfusion chambers, part of the signal is transmitted, and part is reflected back. Then, a coupler guides the reflected signal to a photodetector that collects the intensity data at a 1 kHz sampling rate. Finally, a computer routine programmed in MATLAB (MathWorks) processes the data and converts it into useful information about the cell environment [20].

The reflected signal intensity I_R_ is modulated by the difference between the fiber refractive index, n_1_, and the medium refractive index, n_2_, according to the Fresnel principle, Equation (1), where I_0_ is the intensity of the emitted signal and R is the power reflectance [21].

(1) $$\frac{I_{R}}{I_{0}}=R={\left[\frac{\left(n_{1}-n_{2}\right)}{\left(n_{1}+n_{2}\right)}\right]}^{2}$$

When analyzing the intensity signal of a particulate system, where the particles’ sizes are comparable in order of magnitude to the laser wavelength, it is possible to observe fluctuations in the intensity signal which are related neither to changes on the average sample refractive index nor to the intrinsic uncertainties of the electronic system. This increase in the data dispersion is, in fact, due to the quasi-elastic light scattering (QELS) phenomenon, which consists of a mechanism of energy absorption by the particles followed by a new emission of radiation [22].

This phenomenon can be analyzed by evaluating the autocorrelation function G_2_(τ) of the reflected light intensity I_R_(t) for the obtention of information about the instant concentration of cells. The function G_2_(τ) is defined by Equation (2) [22,23], where τ is an arbitrary delay time.

(2) $$G_{2}\left(\tau \right)=\underset{T\to \infty }{\lim}\frac{1}{T}\overset{T}{\underset{0}{\int }}I\left(t\right)\cdot I\left(t+\tau \right)\mathrm{dt}≅\underset{N\to \infty }{\lim}\frac{1}{N}\overset{N}{\underset{j=1}{\sum }}I\left(j\right)\cdot I\left(j+\tau \right)$$

The decay rate Γ of G_2_(τ) is a function of the translational diffusion coefficient of a particle A in a fluidic medium B, D_AB_, as expressed by Equation (3), where q is the magnitude of the scattering vector (the difference between the emitted light and the reflected light vectors) [22]. The vector q is proportional to the total number of particles scattering the light, so it is possible to correlate Γ to the concentration of cells inside the microchamber.

(3) $$\Gamma =D_{\mathrm{AB}}q^{2}$$

Several mass-transfer models show that the diffusion coefficient is a function of the system’s temperature and of the inverse of the particle’s average diameter [24]. Therefore, the average decay rate Γ_m_ is expected to be lower when working with cells of higher average diameter, and higher rates are expected for experiments conducted under higher temperatures. On the other hand, Equation (2) is a statistical measurement that relies on the total number of particles, so more reliable results are expected for higher concentrations [21].

The G_2_(τ) function experimentally obtained by the FOQELS is related to the average decay rate Γ_m_ by the Siegert relation, Equation (4) [22], where α and β are parameters obtained by fitting Equation (4) to the G_2_(τ) data.

(4) $$G_{2}\left(\tau \right)=\alpha +\beta \cdot \exp\left(-2\Gamma_{m}\tau \right)$$

The computational routine reads the experimental values I_R_(t) and applies them to the algorithm described by Equations (2)–(4), obtaining values of Γ_m_ that will be posteriorly correlated to the instant concentration of cells by a calibration curve.

**Figure 2:**
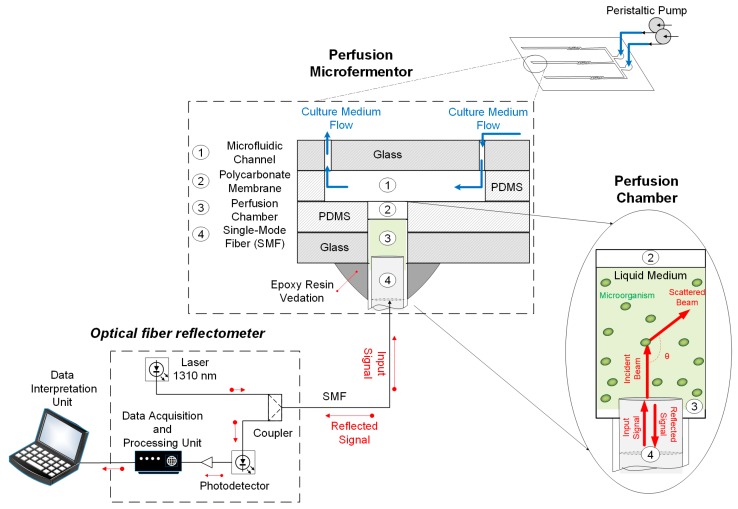
Schematic representation of the instrumented microfermentor. A peristaltic pump flows culture medium through the microchannels of the PDMS top layer and silica single-mode fibers (SMFs) are inserted inside the perfusion chambers, where cells scatter the light (1310 nm laser).

A comprehensive schematic of the instrumented microfermentor and the measurement system is shown in Figure 2. A peristaltic pump flows substrate through the microchannels of the PDMS top layer, and SMFs are inserted inside the perfusion chambers, evaluating the scattering of the laser light by the cells.

### 2.3. Microbial Cultivation, Growth Kinetics, and Experimental Procedure

*Saccharomyces cerevisiae (S. cerevisiae*) ATCC 7754 cells were cultivated in a complex medium known as yeast-peptone-dextrose, YPD, which is comprised of 10 g of yeast extract, 20 g of peptone, and 20 g of dextrose per liter of water, with pH of 6.5 ± 0.2 [25].

The mathematical models for the kinetics of cell growth are based on the definition of the specific cell growth rate μ, Equation (5), where X is the cells’ biomass concentration, and t is the time from the beginning of the fermentation [1,4].

(5) $$\mu =\frac{1}{X}\frac{\mathrm{dX}}{\mathrm{dt}}$$

A mathematical model commonly applied for the correlation of μ to the substrate concentration S, which is able to predict the general kinetic behavior and can be constructed with a relatively low amount of experimental data is the Monod model, Equation (6). This model does not take into account the latency period necessary for the microorganisms to adapt themselves to the cellular environment, so, if this phase is observed, the latency data must not be used for the obtention of the two model parameters, the maximum specific growth rate, μ_m_, and the Monod constant, K_M_ [1,4].

(6) $$\mu =\left(\mu_{m}\right)\frac{S}{K_{M}+S}$$

The parameters obtained are functions of different factors, and the μ_m_ calculated for the alcoholic fermentation of *S. cerevisiae* depends, for example, on the carbohydrate used as a substrate; it is higher for a simpler carbohydrate, such as glucose or fructose than for a more complex carbohydrate, like sucrose [26]. If different carbohydrates are available, the microorganisms firstly process the simpler ones and only then consume the more complexes substrates [1,4]. Consequently, due to these differences and to the constant adaption of the microorganisms to the environmental conditions, small deviations on the constants’ values may be observed even when simple changes such as the use of different reactional volumes are performed, so the parameters should be routinely recalculated. The parameters found in literature, in their turn, must be applied as first approximations for the models and for the comparison of the final results. We took the values of μ_m_ = 0.42 h^−1^, and K_M_ = 4.10 g/L as the initial parameters for the fermentation of *S. cerevisiae* under 33 °C, which were obtained for a complex substrate similar to YPD [27].

#### 2.3.1. Definition of Experimental Conditions

The Equation (6) was analyzed for the values chosen for the Monod parameters, resulting in ~43% of the total expected variation of μ for concentrations S inferior than 2.5 g/L. However, in industrial fermentation processes of *S. cerevisiae*, substrate concentrations are on the order of 131–224 g/L [26,28]. Some typical values of substrate concentrations are 50 to 200 g/L of glucose [29]; 100 g/L of sucrose [30]; 10 g/L of glucose [31]; 1.6–5 g/L of sugarcane molasses [32]; and 121.7–222.1 g/L of total sugars quantified from molasses [33].

Therefore, it is not interesting for practical purposes to test S values lower than 2.5 g/L and, in such low substrate concentrations, the verified kinetics could be due to the metabolization of the substances remaining in the YPD medium, which contains dextrose and nitrogen compounds that stimulate the cellular growth [25]. Then, a reliable correlation would not be obtained for such low concentrations, so the sucrose concentrations of 2.5, 5.0, 10.0, 15.0, 20.0, 25.0, and 30.0 g/L were chosen to flow into the microchannels for the sensor tests (temperatures kept constant in 33 °C). From the resolution of Equation (6) with the chosen initial Monod parameters, and considering the low volume of the microchambers (consequently, the low total number of cells inoculated) the value of 30 g/L is already a large excess of substrate, so no higher concentrations of S were evaluated.

#### 2.3.2. Obtention of the Calibration Curve and the Batch Kinetics

Cells were inoculated in YPD medium and grown at 33 °C overnight. The cells were progressively diluted in ultrapure water (Purelab Option-Q, 18.2 MΩ·cm, Elga Veolia, High Wycombe, England) and tested with the optical fiber sensor before the insertion and sealing of the fibers into the microfermentor.

The tests were repeated for the temperatures of 25, 30, and 35 °C—the variation of temperature usually observed in bioprocesses [4,27]—for the verification of the need of temperature corrections on Γ_m_ related to the increase of the particles’ diffusivities [24], and all of the experiments were performed in triplicate.

The cells were also observed on an optical microscope using 10× magnification (Nikon Eclipse Ti, Nikon, Tokyo, Japan) for their visual counting with the Neubauer chamber procedure (a traditional technique applied for the evaluation of concentrations of cells, but with uncertainties on the order of 20–30% [34]). This full process, which allows the correlation of Γ_m_ to the concentration of cells in the number of cells/mL, is extensively detailed in the Supplementary Material.

It is imperative to compare the sensor performance with a traditional method already used to obtain the growth kinetics, e.g., the evaluation of the growth in a batch reactor monitored with the microscope. For this control experiment, a microbiological handle was used for removing a small sample of cells from the saturated culture medium, and then the handle containing the cells was introduced and used for the agitation of 25 mL of fresh YPD medium previously sterilized for the inoculation of the cells. The solution was kept at 33 °C and 100 rpm rotation for 10 h and, every hour, a 1 μL sample was removed from the medium and used for the cell counting with the Neubauer chamber on the microscope. 

It is important to notice that the Monod equation is not adequate for the analysis of this comparative experiment since the exact concentration of substrate is not known. Then, a procedure adequate for the evaluation of μ_m_ in this particular case is the fitting of the experimental data to a simpler logistic model that does not take into account the substrate concentration, and that presents satisfactory results for fermentations like this one, where there is a large excess of the substrate [35]. The discussion of this simpler mathematical model and the procedure for obtaining μ_m_ in this particular test—a result that is important for the evaluation of the sensor performance—are given as Supplementary Material.

#### 2.3.3. Perfusion Microfermentor Tests

We observed a latency period of ~5 h for the batch experiment (more details in the Results section and Supplementary Material). Thus, it was decided to preheat the YPD medium saturated with cells at 33 °C and atmospheric air for 5 h before the inoculation. The interval was considered sufficiently long for the medium to reach the solubility equilibrium of oxygen under the experimental conditions and, therefore, to ensure dissolved oxygen for the cells. 

The medium was diluted in water (50:50 *v/v*) and introduced in the microfermentor. This dilution is important for the analysis of the growth inside the microdevice and for testing the sensor performance. If we start with a high concentration, there will be a low variation of the concentration before the chamber becomes saturated in cells and, once the sensor presents an intrinsic sensitivity (Section 3), this variation may be very low for the sensor to give significantly different signal results. 

After the inoculation of cells, a peristaltic pump (MPS 380, Marte Científica, São Paulo, SP, Brazil) was connected to the microreactor to pump different concentrations of sucrose (2.5 to 30 g/L) within the microchambers (flow rate fixed in 9 μL/min). In each experiment, the same concentration S was applied to both entrances, leading to results obtained in triplicate.

The fermentations were monitored for 4.5 h, and the calibration curve was applied for the conversion of Γ_m_ in concentration. The parameters calculated for the microorganisms (μ_m_ and K_M_) were the ones which best represented all of the eight experiments, so a Microsoft Excel Visual Basic for Applications (VBA) optimization algorithm was designed for the calculus of the parameters. It consists of a simple try-and-error algorithm that keeps the value of K_M_ constant and equals to 4.2 g/L (once this parameter is less prone to temperature and other environmental variations [27]); varies μ_m_ from 0.60 to 0.10 h^−1^ (an interval that contains the values typically found in the literature for *S. cerevisiae*, which vary from 0.17 to 0.50 h^−1^ [36,37]) with steps of 0.01 h^−1^; calculates the errors, i.e., the differences between the experimental values of the concentration and the correspondent values that are obtained by numerically solving Equations (5) and (6) with the 4th Order Runge–Kutta Method (step of 0.1 h) for K_M_ and for the tested μ_m_; calculates the square of each error and the sum of all the error squares; chooses the parameter μ_m_ that results in the lowest value of the sum of the squares (adjustment by the minimization of error squares); and solves Equations (5) and (6) for the selected parameters.

A final comparative experiment was performed with a microfermentor without the optical fibers and submitted to the concentration of sucrose of 30 g/L (condition of excess of substrate, allowing the comparison with both the batch experiment and the microdevice with the optical fiber sensor). The assay was monitored by microscopy, and its major importance was to obtain visual confirmation of the presence of the cell growth inside the microchambers. 

Once the yeast cells present low diffusion coefficients due to their high diameters [24], and suffer no chemotaxis, remaining in approximately the same spatial position during the experiment [2,38], it is possible to define isolated virtual regions of the chambers with a uniform distribution of cells where the growth is further analyzed. Therefore, 200 × 200 μm^2^ square areas were delimited, and the heights of these chambers were considered equal to 0.50 mm (approximate thickness of the PDMS layer), resulting in virtual volumes of 2 × 10^−5^ mL. The micrographs obtained for these regions were analyzed by a MATLAB image processing routine which identifies the particles’ centroids inside the virtual volumes, counts the total of the centroids and evaluates each diameter in a pixel scale. A critical diameter must be manually defined for allowing the routine to differentiate cells from aggregates and, if there are aggregates present, they are not counted, and the routine advises the user not to apply that particular region for the growth evaluation.

All of the experiments and microbial cultures described in this research were performed in accordance with the rules and standards of the Bioethical Committee of the University of Campinas, and the project was declared to the Brazilian’s Genetic Heritage Database, according to the laws enforced in Brazil.

## 3. Results and Discussion

**Figure 3:**
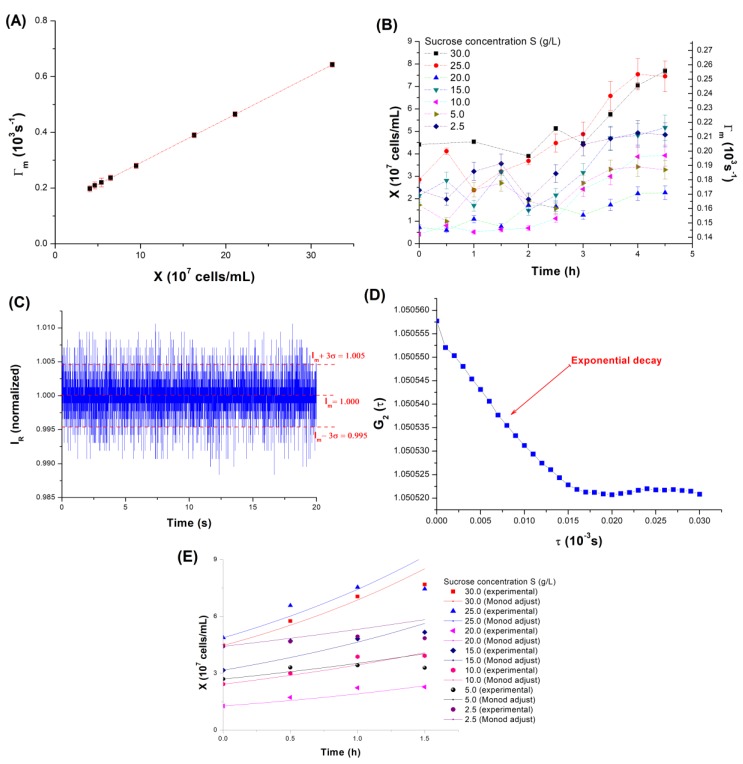
(**A**) Correlation between Γ_m_ and the concentration of cells; (**B**) concentrations experimentally obtained with the microfermentor for each concentration of sucrose in the microchannels, with the Γ_m_ values shown on the secondary axis; (**C**) normalized optical intensity I_R_ corresponding to the highest verified Γ_m_ (30 g/L of sucrose, t = 4.5 h), and its interval I_m_ ± 3σ; (**D**) autocorrelation function G_2_(τ) obtained from the signal of Figure 3C, showing the decay characteristic of the quasi-elastic light scattering (QELS); (**E**) comparison between experimental results of the microfermentor and the curves obtained with the Monod equation solved for the parameters calculated by the Visual Basic for Applications (VBA) algorithm (K_M_ = 4.1 g/L and μ_m_ = 0.49 h^−1^).

Figure 3A shows the correlation between Γ_m_ and the cells concentration. We did not observe differences on Γ_m_ regarding the evaluated temperature range (25–35 °C), indicating that no temperature compensations were needed, so the results obtained for the same concentration were used as replicates.

The sensor response increases linearly with the concentration. Equation (7) shows the linear curve fitting with the correlation coefficient R^2^ of 0.995, indicating a good agreement between the experimental data and the model. In this equation, Γ_m_ is expressed in units of 10^3^ s^−1^, X is given in the number of cells per mL, and the fitting is valid for the 4–35 × 10^7^ cells/mL range.

(7) $$\Gamma_{m}=\left(1.56159\times 10^{-9}\right)X+0.13563$$

Since the optical signal is a function of the refractive indexes difference (Equation (1)) and of the laser wavelength [21], it is necessary to determine particular calibration curves for different light sources because the scattering may not be verified if the particles diameters and the wavelength are not comparable in magnitude [22].

The sensor sensitivity was calculated as the rate of variation of the signal to the concentration of cells, (dΓ_m_/dX) [21] by simply deriving the curve fitting, yielding a 1.56159 × 10^−6^ mL·s^−1^·(N_c_)^−1^ sensitivity, where N_c_ was included as a representation of the number of cells, but was, in fact, dimensionless.

Methylene blue was used for verifying the presence of cellular death (that could be caused, for example, by the shear stresses imposed to the YPD when this medium was diluted in water), but no dead cells could be distinguished with the optical microscope.

It is known that the addition of sugars (such as sucrose) enhances the refractive index of water and then modulates the optical signal (Equation (1)), but it was not possible to verify significant changes in the power reflectance R. This can be explained by the low concentrations of dextrose on the progressively diluted YPD medium, once the mass fractions of sucrose lower than 10% are not expected to lead to significant reductions on the reflected intensity [20].

The batch fermentation curve, the images applied for the cell counting, and the fitting of the data to the logistic model (resulting in an adjusted R^2^ of 0.859) are shown in the Supplementary Material, in Figures S3 and S4. These results lead to an estimated value of μ_m_ = 0.50 h^−1^ for the batch growth with excess of substrate, which is consistent for microorganisms with optimized protein complexes, and with fast growth in conditions of excess of substrate [1,4,27]. A latency period of approximately 5 h was verified before the effective cell growth, so it was necessary to preheat the cells under 33 °C (Section 2.3.3) before the microfermentor experiments.

Figure 3B shows the concentrations measured for each experiment with the microfermentor, with the concentration of cells shown on the main axis, and the value of Γ_m_ obtained by the optical system shown on the secondary axis. The dilution before the inoculation in the microdevice allowed the verification of an effective growth, but a shorter latency period of ~3 h was observed for all experiments. This is probably related to the adaption of the cells to the different environment of the microfermentor before growing [4].

Once the data dispersion was increased by the light scattering, the highest Γ_m_ value (test with 30 g/L of sucrose at t = 4.5 h), was chosen for estimating the maximum signal to noise ratio (SNR). However, it is important to note that in case of the QELS, higher SNRs correspond to higher Γ_m_, i.e., to an increase in the sensitivity concerning the concentration measurements.

The normalized intensity I_R_ for this case is shown in Figure 3C, and the SNR was calculated as the ratio I_m_^2^/σ^2^, where I_m_ is the average value and σ is the standard deviation of the signal [21], resulting in an SNR = 4.169 × 10^5^. The same information can be interpreted in terms of the interval I_m_ ± 3σ, taken as an estimate of the total uncertainty of the signal, yielding 1.000 ± 0.005 in this case. The other experiments provided lower SNRs and narrower I_m_ ± 3σ intervals due to the lower number of dispersed cells. Figure 3D shows the autocorrelation function G_2_(τ) calculated from the signal of Figure 3C and exhibits the exponential decay characteristic of QELS (Equation (4)), which allows the calculation of Γ_m_.

By removing the latency period of 3 h, it is possible to find the Monod parameters using the algorithm described in the previous section, resulting in K_M_ = 4.1 g/L and μ_m_ = 0.49 h^−1^ by the minimization of the squares of the errors. The comparison between the experimental data obtained with the microfermentor and the kinetic theoretical curves (numerical solutions of the Monod equation) calculated with these parameters are shown in Figure 3E. 

By comparing the obtained parameters with the values reported by other authors, Table 2, one may notice that the kinetic behavior verified with the microfermentor (for the total of eight experiments) corroborates with the batch experiment of 25 mL, showing a difference of only 2%. The deviations may be explained by the uncertainty of the visual procedure (20–30% [34]), by the fact that more tests were performed with the optical sensor, by the modification on both the operation mode and the volume of the fermentation broth, and by the possibility of temporal evolution of the microorganisms throughout the successive growth cycles [10,13]. Another hypothesis is that, due to the very low volume of the microfermentor (~0.01 mL in each perfusion chamber), it is easier for the cells to adapt and saturate the medium, resulting in a slightly lower total growth and, consequently, a lower μ_m_ value.

On the other hand, Table 2 shows that both results are in accordance with those verified in the literature, where values of μ_m_ ranging from 0.17 to 0.50 h^−1^ and K_M_ ranging from 0.099 to 4.56 for the fermentation with *S. cerevisiae* are found. The possible discrepancies in the reported values are due to the differences in the strains of microorganisms, monitoring and fermentation strategies, and used substrates. In fact, the value calculated with the microfermentor is equal to that achieved by Postma et al. (2000) [43], whereas that obtained in the batch test is the same as that verified by Jain (1970) [36].

**Figure 4:**
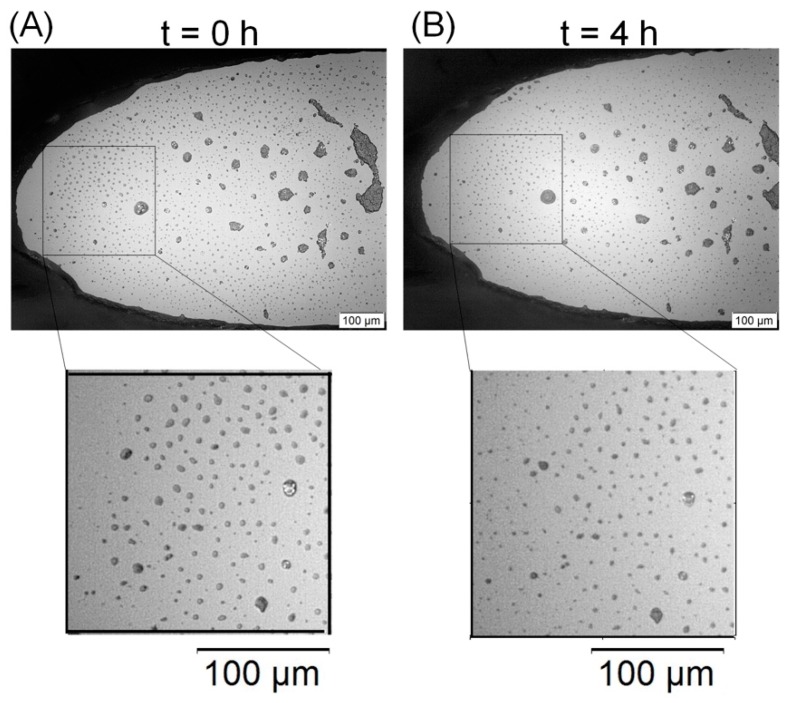
Square regions (2 × 10^−5^ mL) used for the visual estimation of the cell growth, for two different times: (**A**) t = 0 h; (**B**) t = 4 h. It is possible to observe in the inferior details of the increase in the number of dark points (left side of the square), which represents the *S. cerevisiae* cells.

Finally, the presence of cellular growth inside the microchambers for the sucrose concentration of 30 g/L was proven with the microscope. Figure 4 shows one of the volumes of 2 × 10^−5^ mL chosen for cells counted at t = 0 and t = 4 h, when this test was finished.

**Figure 5:**
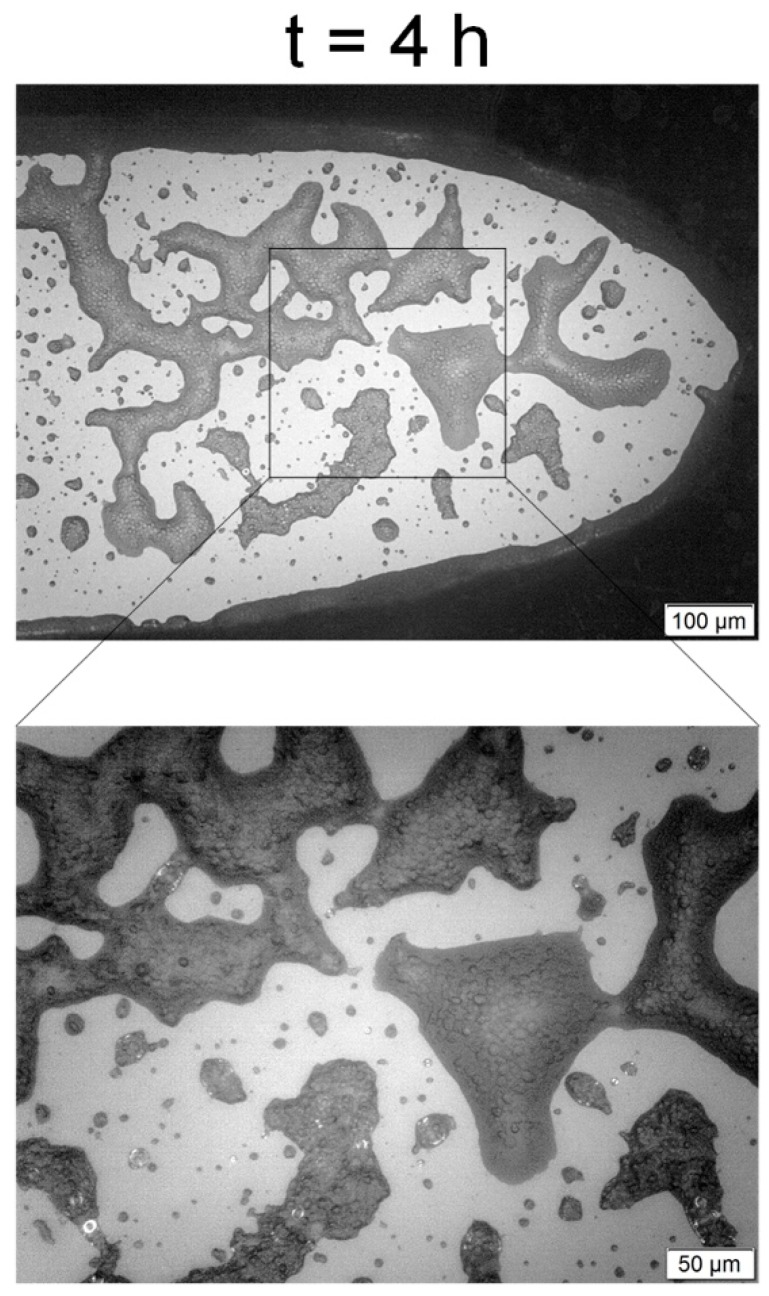
Region with formation of cell colonies under two magnifications, 10× in the superior image and 20× in the inferior image, allowing the observation of the cells’ boundaries and almost spherical morphology.

Figure 5 shows a region with an agglomeration of cells and formation of colonies in one of the microchambers after 4 h, which is not adequate for the cell quantification due to both the difficulties inherent to the counting procedure and the non-homogeneity of the local concentration of cells. Figure 4 was obtained using a lens with a magnification of 10×, whereas Figure 5 was obtained with two different magnifications, 10× for the superior image and 20× for the more inferior image (for more details). Besides the nominal magnification of the lenses, the microscope camera also presented a second magnification of 10×.

Figure S5 of the Supplementary Material shows the comparison between the cell concentrations observed during the first 4 h for the three methodologies. It is possible to observe similar shapes with an increasing tendency for the three curves, and also the same order of magnitude for the estimation of the cell concentration. 

However, the fermentation monitoring results obtained with the microscope were the least trusted of them as shown in Figure 5, where it is possible to observe regions in the microfermentor with the formation of colonies, which present higher local concentrations. Due to the tridimensional geometry of these colonies and to the bidimensional observation of the microscope, it is not possible to obtain the precise concentration of cells in these regions and, if these cells were homogeneously distributed throughout the microchamber, the counting results on the areas defined for growth evaluation would be probably quite superior. There are also errors associated with the volume estimation, which did not take into account the contraction of the PDMS material due to the adhesion with the glass or due to the pressure exerted by the acrylic molds. There is also the hypothesis that the cells stay fixed in a given position of the microchamber, which does not take into account neither the Brownian motion related to the diffusion nor the cells divisions. The FOQELS, on the other hand, does not present those problems, as the decay rate is a statistical property of the whole medium surrounding the fiber end face and is related to the diffusivity of the particles.

Therefore, the main importance of Figure 4 and Figure 5 is the visual verification of the presence of the microbial growth inside the chambers and the observation of the cells’ approximate spherical morphologies, with diameters comparable to the laser wavelength. These results, combined with the discussion of Table 2, demonstrate the efficiency and validity of the sensor for fermentation monitoring and for the screening of the growth parameters.

## 4. Conclusions

We demonstrated a reversible microfermentor integrated to an optical fiber sensor that allows the fast screening of cell growth kinetic parameters, resulting in K_M_ = 4.1 g/L and μ_m_ = 0.49 h^−1^ for *S. cerevisiae* cells, values that are in agreement with the literature. These results were compared to the traditional batch experiments for kinetic evaluation, showing data consistency (difference of 2% in the calculated parameters), and the growth inside the microchambers was also empirically verified with the microscope. The perfusion chambers proved to be very useful—they are able to keep the substrate concentration constant while allowing the removal of products and metabolites—which not only facilitates the numerical simulations of the Monod equation, but also protects the microorganisms against the lack of nutrients and accumulation of toxic substances.

The proposed sensor is an alternative to visual procedures, and the quality of the results could be further improved by the correlation of the signals to the dry masses of cells. It would be performed by analyzing solutions with unknown cellular biomasses with the optical fiber system, and then taking samples with known volumes from those media for the centrifugation, allowing the comparison between solid masses’ (cells) concentrations and signals. This methodology, on the other hand, can only be applied to biosystems where the mass accumulation is directly proportional to the cellular growth, which is not verified for filamentous fungi, for example [13,25].

## Figures and Tables

**Table 1:** Mathematical symbols given in the order they are introduced in this research.

Symbol	Meaning	Units
X	Concentration of Cells	(number of cells)·mL^−1^
S	Concentration of Substrate (e.g., Sucrose)	g·L^−1^
I_R_	Normalized Reflected Intensity Signal	-
I_0_	Normalized Reference Signal	-
R	Power Reflectance	-
n_i_	Refractive Index of Medium “i”	-
G_2_	Autocorrelation Function of I_R_	-
Γ	Decay Rate of the Autocorrelation	s^−1^
t	Instant of the Measurement	s or h
τ	Arbitrary Delay Time	s
D_AB_	Diffusivity of Species “A” on Medium “B”	cm^2^·s^−1^
q	Light Scattering Vector	cm^−1^
α and β	Fitting Parameters	-
μ	Specific Growth Rate	h^−1^
μ_m_	Constant of Maximum Specific Growth Rate	h^−1^
K_m_	Monod Constant	g·L^−1^

**Table 2:** Comparison between growth parameters obtained by the two methodologies to those reported in other researches.

μ_m_ (h^−1^)	K_M_ (g/L)	Reference
0.49	4.1	(obtained with the microfermentor)
0.50	-	(obtained with the batch fermentation)
0.42	4.1	[27]
0.50	0.187	[36]
0.17	0.5	[37]
0.32	0.27	[38]
0.24	4.56	[39]
0.40	1.51	[40]
0.42	0.50	[41]
0.31	0.099	[42]
0.49	3.6	[43]

## Supplementary Materials

The following are available online at https://www.mdpi.com/1424-8220/19/11/2493/s1, Figure S1: Neubauer chamber containing 0.143 C, where C is the cell concentration on the yeast-peptone-dextrose (YPD) medium saturated on yeast cells. It is possible to notice tridimensional agglomerations. Figure S2: Five squares of the Neubauer chamber used for the cell counting on a suspension with concentration of 7.69 × 10^−4^ C, where C is the concentration of cells on the saturated yeast-peptone-dextrose (YPD) medium. The analyzed squares and the counted cells are highlighted in red. Figure S3: Cell concentration profile over time for 10 h and the images of the Neubauer chamber obtained (images of 3 different times). Figure S4: Fitting of Equation (S3), used for the estimation of the maximum specific growth rate, μm, compared to the experimental data. Figure S5: Comparison between batch results, results collected with the optical fiber sensor (OFS) experiment (sucrose concentration of 30 g/L), and results obtained by microscope observations for the first 4 h of each experiment (30 g/L of sucrose).
