# Supplementary material for: Perfusion Microfermentor Integrated into a Fiber Optic Quasi-Elastic Light Scattering Sensor for Fast Screening of Microbial Growth Parameters
Source: Sensors (Basel). 2019 May 31;19(11):2493. doi: 10.3390/s19112493 (PMC6603560; doi:10.3390/s19112493)
Supplement: Supplementary file 1 [file sensors-19-02493-s001.pdf]

sensors

Article

Perfusion Microfermentor Integrated into a Fiber Optic Quasi-Elastic Light Scattering Sensor for Fast Screening of Microbial Growth Parameters

Marco César Prado Soares [1,*], Franciele Flores Vit [2], Carlos Kenichi Suzuki [1], Lucimara Gaziola de la Torre [2] and Eric Fujiwara [1]

[1] Laboratory of Photonic Materials and Devices, School of Mechanical Engineering, University of Campinas, São Paulo, 13083-860, Brazil; suzuki@fem.unicamp.br (C.K.S.); fujiwara@fem.unicamp.br (E.F.)
[2] Laboratory of Advanced Development of Nano and Biotechnology, School of Chemical Engineering, University of Campinas, São Paulo, 13083-852, Brazil; franciele.floresvit@gmail.com (F.F.V.); ltorre@g.unicamp.br (L.G.d.l.T.)
* Correspondence: marcosoares.feq@gmail.com; Tel.: +55-19-3521-3337

Received: 23 April 2019; Accepted: 27 May 2019; Published: 31 May 2019

S1. Cell Counting in the Neubauer Chamber for Sensor Calibration

Yeast-Peptone-Dextrose (YPD) medium saturated on *Saccharomyces cerevisiae* ATCC 7754 cells with unknown concentration C was progressively diluted in milli-Q water (Purelab Option-Q, 18.2 MΩ.cm, Elga Veolia, High Wycombe, England) and all of the suspensions were evaluated using the fiber optic quasi-elastic light scattering sensor (FOQELS) in order for obtaining the calibration curve between the autocorrelation decay rates and the cell concentrations. Figure S1 shows the Neubauer chamber containing a concentration of 0.143 C, where C is the concentration of cells in the saturated medium (lens with magnification of 10×).

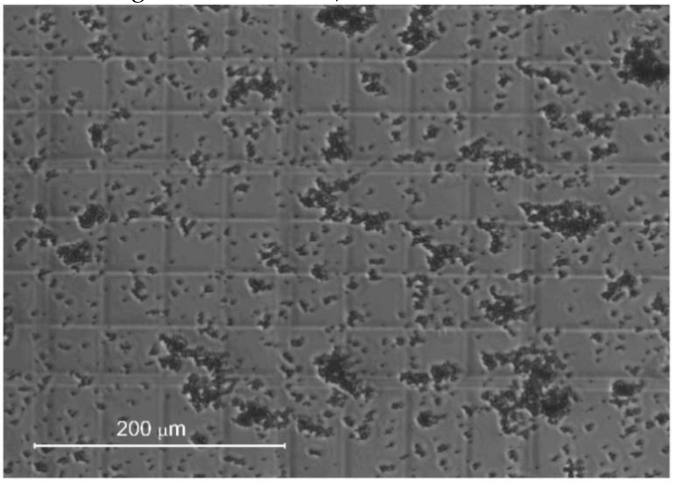

Figure S1. Neubauer chamber containing 0.143 C, where C is the cell concentration on the yeast-peptone-dextrose (YPD) medium saturated on yeast cells. It is possible to notice tridimensional agglomerations.

Due to the relatively high concentration, it is possible to notice tridimensional agglomerations of cells, what makes Figure S1 inadequate for the cell counting. Then, the medium must be further diluted before the evaluation of the concentration of cells. If a sample with volume V of saturated medium is diluted in water, then the concentration C_2 of the diluted medium after mixing (total volume V_2) will be related to the concentration C and to the sample volume V by Equation (S1), which is a direct consequence of the conservation of mass (the mass must be the same at the beginning and at the end of the dilution process).

$$C_2 = \frac{CV}{V_2} \quad \text{(S1)}$$

The Neubauer chamber is divided in square regions with defined volumes. The squares present different areas, in order for making it possible to evaluate different types of cells, but present the same height, 0.1 mm. For the evaluation of yeast cells, it is possible to use the squares with 16 subdivisions and volumes of 4×10^{-6} mL.

The experimental concentration will be given by the number of cells divided by the volume used for the analysis. If the dilution is not very high, then the distribution of cells inside the chamber is approximately homogeneous and it is possible to take the average value obtained for individual squares as the concentration. On the other hand, if the concentration is too low, the distance between individual cells is very high and the distribution inside the chamber is not homogeneous, with squares containing no cells. In this case, it is necessary to sum different squares and to divide the counting by the total volume of analysis.

It is interesting to note that the results can be improved by adding a small amount of methylene blue dye solution (5 mg of methylene blue/ L of water) as a cell viability analyzer: the dead cells are permeable to this dye and are seen as dark objects on the microscope. It is then possible to count only the viable cells [1,2].

For the calibration of the sensor, the five squares of the main diagonal of the chamber were used, totalizing a volume of 2×10^{-5} mL. 500 μL of the methylene blue solution were added to 800 μL of a suspension with concentration $C_2 = 0.125$ C, and 1 μL of this solution was mixed to 99 μL of milli-Q water. Then, this final suspension (concentration of 7.69×10^{-4} C) was analyzed in the Neubauer chamber using an objective lens with magnification of 10×, as shown on Figure S2, where the counted cells and the analyzed squares are highlighted in red.

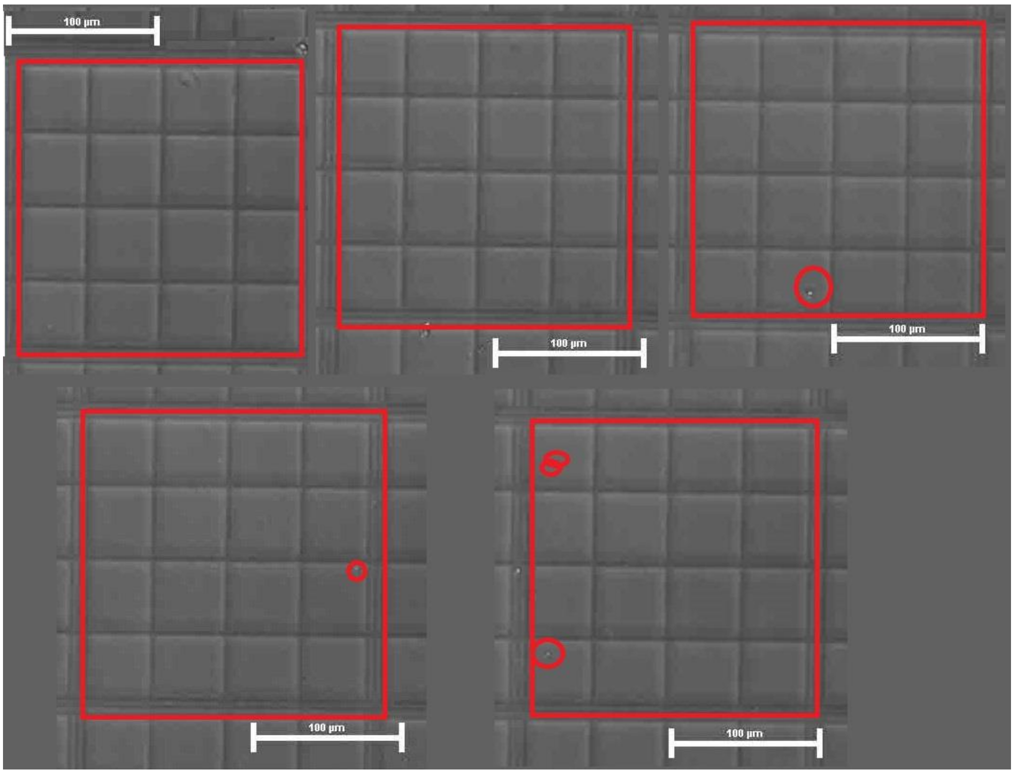

Figure S2. Five squares of the Neubauer chamber used for the cell counting on a suspension with concentration of 7.69×10^{-4} C, where C is the concentration of cells on the saturated yeast-peptone-dextrose (YPD) medium. The analyzed squares and the counted cells are highlighted in red.

The cell counting resulted in concentration of cells on the YPD saturated medium of C = 3.25×10^{8} cells/mL. It is important to notice that the Neubauer chamber manufactures guarantee an uncertainty of 20–30% for this procedure (i.e., uncertainty of 9.75×10^{7} cells/mL) [3].

Then, suspensions progressively diluted, with concentrations of C, 0.650 C, 0.500 C, 0.300 C, 0.200 C, 0.167 C, 0.143 C and 0.125 C were evaluated with the FOQELS, resulting in the calibration curve.

S2. Batch Fermentation Analysis

Saccharomyces cerevisiae ATCC 7754 cells were inoculated in 25 mL of YPD medium previously sterilized using a microbiological handle, and the system was kept under 33 °C and 100 rpm rotation for 10 h. Every hour, a sample was collected from the fermentation broth and introduced into the Neubauer chamber, being posteriorly analyzed in the microscope. In this experiment, the cells were homogeneously distributed inside the chamber, so the average concentration inside each one of the 5 squares of the main diagonal of the Neubauer chamber was took as the real concentration for a given time and the standard error ($s/5^{0.5}$, where s is the standard deviation) was took as the uncertainty.

The first sample of 1 μL was not diluted; the samples of 10 μL corresponding to the times of 1 h to 6 h were mixed with 90 μL of milli-Q water (dilution of 10 times) before the analyses; the other samples, of 1 μL, were mixed with 99 μL of milli-Q water (dilution of 100 times) before the analyses.

Defining N as the number of cells per mL of analyzed volume, the kinetic curve can be obtained (Figure S3). It is possible to notice a sigmoidal behavior, with an initial latency followed by a fast exponential growth and a final baseline. Figure S3 also shows the cells inside the chamber for three different times (images obtained using microscope lens with magnification of 10×).

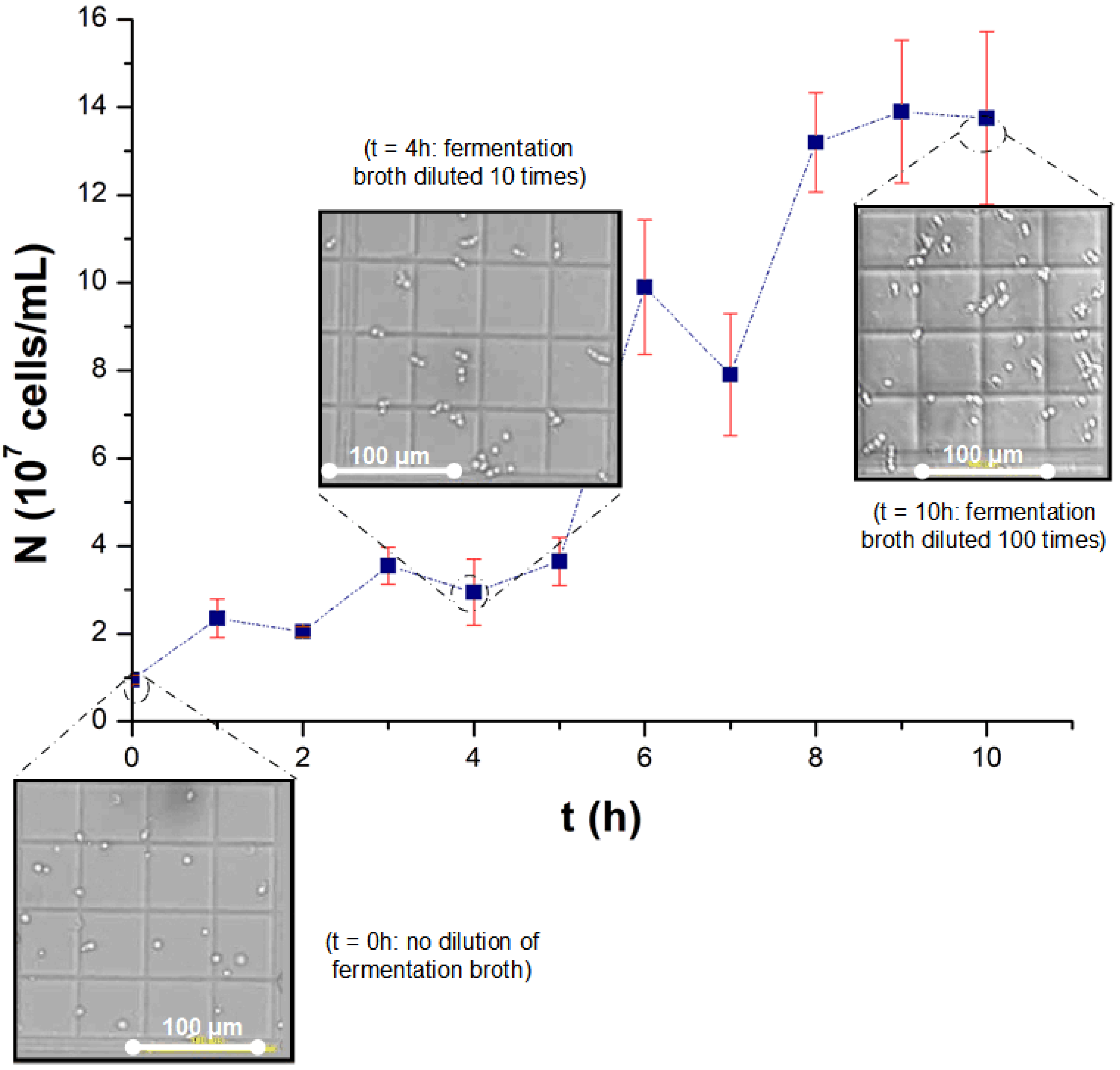

Figure S3. Cell concentration profile over time for 10 h and the images of the Neubauer chamber obtained (images of 3 different times).

A first important consideration is the presence of a latency phase of approximately 5 h before the fermentation reaches the exponential growth, which is related to the protein complexes of the microorganisms and to their adaption to the fermentation environment [1,2]. Due to this result, the fermentation broth was kept under 33 °C for 5 h before the analysis of the other experiments, favoring the observation of the cell growth and the detection by the optical system. Another remarkable fact is that the exponential growth phase is very fast, with ~2 hours of duration.

Since the fermentation broth used in this experiment consists of a complex medium with great excess of substrates, the microbial growth can be evaluated in terms of a sigmoidal model, Equation (S2) [4]. The parameter N_m represents the maximum concentration of cells, the final baseline, and μ_m is the maximum specific growth rate.

$$\frac{dN}{dt} = \mu_m N\left(1 - \frac{N}{N_m}\right) \quad \text{(S2)}$$

Equation (S2) can be manipulated for the separation of variables, and then it can be integrated, leading to Equation (S3) [4], where $N_0 = N(t = 0\ h)$.

$$N(t) = \frac{N_0 N_m}{N_m - N_0 + N_0 \exp(\mu_m t)} \exp(\mu_m t) \quad \text{(S3)}$$

The fitting of experimental data to Equation (S3) allows the estimation of μ_m without the precise knowledge of the substrate concentration. Assuming $N_0 = N(t = 0h) = 9.5 \times 10^6$ cells.mL^{-1}, and N_m equals to the average value of the points of the final plateau obtained on Figure S3, $N_m = 1.36 \times 10^8$ cells.mL^{-1}, the regression results in the maximum specific growth rate $\mu_m = 0.50$ h^{-1} and a corresponding adjusted R^2 of 0.859. The comparison between the results obtained by the logistic fitting and the experimental data is shown on Figure S4.

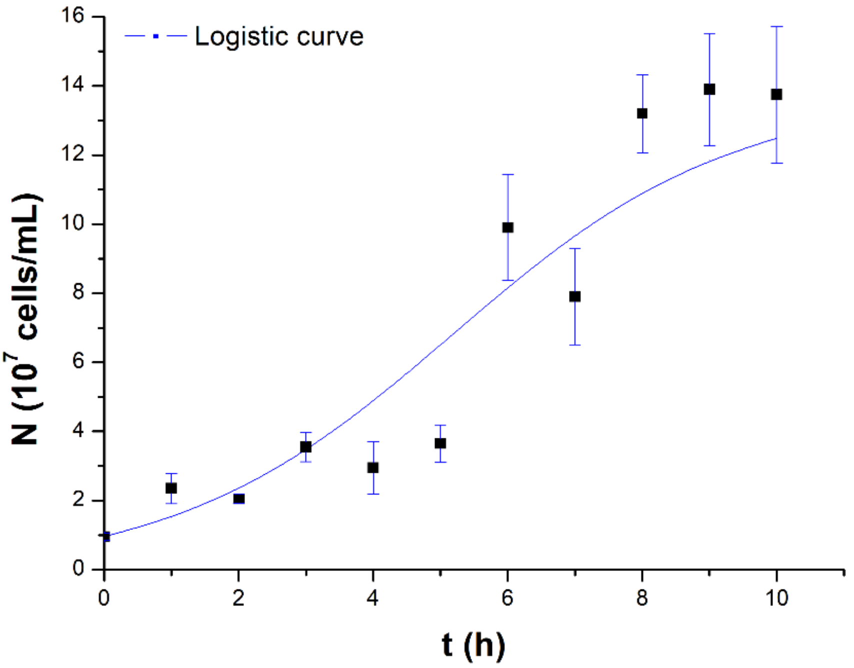

Figure S4. Fitting of Equation (S3), used for the estimation of the maximum specific growth rate, μ_m, compared to the experimental data.

The estimated value of μ_m is in accordance with the reported in literature for this microorganism (Atala et al. reported $\mu_m = 0.42$ h^{-1} for *S. cerevisiae* fermentation under 33 °C using a sugar substrate that offers more difficult to process than either the dextrose of YPD medium or the sucrose, naturally leading to a lower value of maximum specific rate [5], while Sonnleitner and Käppeli [6] and Amillastre et al. [7] reported 0.50 h^{-1}). It is also important to take in account again that the manufacturers of Neubauer chambers guarantee a great uncertainty for this procedure, in the order

of 20–30% of the results [3], and that the exponential growth phase was very fast, so few points could be collected during this period.

S3. Comparison Between Experiments

The results comparing the batch experiment, the test using the microfermentor with sucrose concentration of 30 g/L and the optical fiber sensor, and the observations of the microfermentor on the microscope, without the optical fibers, are shown on Figure S5 for the first 4 h of each experiment.

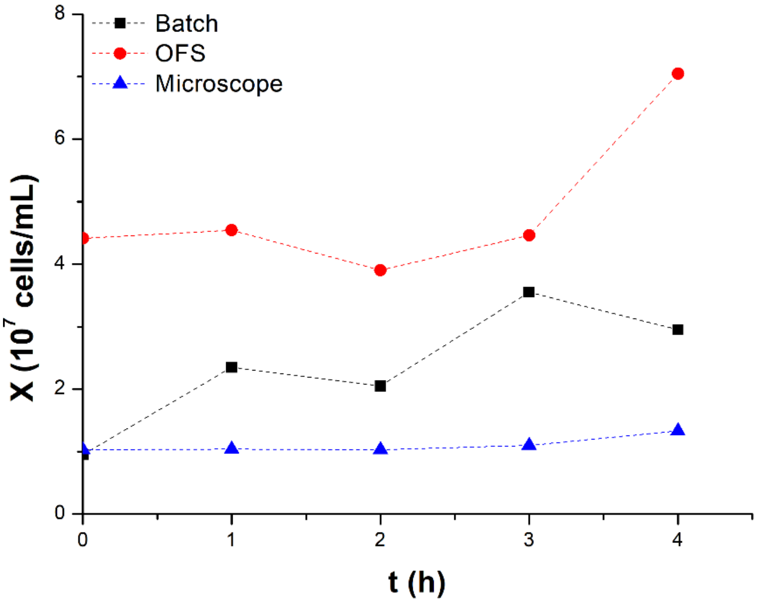

Figure S5. Comparison between batch results, results collected with the optical fiber sensor (OFS) experiment (sucrose concentration of 30 g/L), and results obtained by microscope observations for the first 4 h of each experiment (30 g/L of sucrose).

References

1. Bailey, J.; Ollis, D. *Biochemical Engineering Fundamentals*, 2nd ed; McGraw-Hill: New York City, NY, USA, 1986.
2. Doran, P. *Bioprocess Engineering Principles*, 2nd ed; Elsevier: Amsterdam, The Netherlands, 2013.
3. Celeromics. Cell Counting with Neubauer Chamber, Basic Hemocytometer Usage. Available online in: http://www.celeromics.com/en/resources/docs/Articles/Cell-counting-Neubauer-chamber.pdf (accessed on 14 March 2018).
4. Liu, J.Z.; Weng, L.P.; Zhang, Q.L.; Xu, H.; Ji, L.N. A mathematical model for gluconic acid fermentation by Aspergillus niger. *Biochem. Eng. J.* **2003**, *14*, 137–141.
5. Atala, D.I.P.; Costa, A.C.; Maciel Filho, R.; Maugieri Filho, F. Kinetics of ethanol fermentation with high biomass concentration considering the effect of temperature. *Appl. Biochem. Biotechnol.* **2001**, *91–93*, 353–364.
6. Sonnleitner, B.; Käppeli, O. Growth of *Saccharomyces cerevisiae* is controlled by its limited respiratory capacity: Formulation and verification of a hypothesis. *Biotechnol. Bioeng*. **1986**, *28*, 927–937.
7. Amillastre, E.; Aceves-Lara, C.-A.; Uribelarrea, J.-L.; Alfenore, S.; Guillouet, S.E. Dynamic model of temperature impact on cell viability and major product formation during fed-batch and continuous ethanolic fermentation in *Saccharomyces cerevisiae*. *Bioresour. Technol.* **2012**, *117*, 242–250.

© 2019 by the authors. Submitted for possible open access publication under the terms and conditions of the Creative Commons Attribution (CC BY) license (http://creativecommons.org/licenses/by/4.0/).
